# Catatonia as a Result of a Traumatic Brain Injury

**DOI:** 10.1155/2024/5184741

**Published:** 2024-03-06

**Authors:** Jessica Berthelot, Jacob Cambre, Madeline Erwin, Jennifer Phan

**Affiliations:** ^1^Department of Psychiatry, LSU School of Medicine, 5246 Brittany Drive, 3rd floor, Baton Rouge 70808, LA, USA; ^2^Sciences Center Medical School, Louisiana State University Health, New Orleans, LA 70112, USA; ^3^Department of Psychiatry, University of Illinois at Chicago, 912 South Wood Street, Chicago 60612-7327, IL, USA

## Abstract

Catatonia is a neuropsychiatric syndrome typically marked by disturbances in motor activity, speech, and behavior. It has historically been associated with psychiatric illness, but acute medical illness, neurocognitive disorders, and neurodevelopmental disorders can cause catatonia as well. Catatonia is likely underrecognized and underdiagnosed in the general medical hospital, despite high risks of morbidity and mortality and the availability of rapidly effective treatment. Here, we present a case of catatonia secondary to traumatic brain injury that responded to lorazepam after a delayed diagnosis. A young male patient who was incarcerated and assaulted was sent to the emergency department multiple times for unresponsive and unpredictable behavior, including not agreeing to be released home. After being admitted with the diagnosis of postconcussive syndrome, he was ultimately diagnosed with catatonia, and intravenous lorazepam resulted in a return to his baseline mental status. We discuss factors that led to the delay in diagnosis, including lack of training in recognition of catatonia, suspicion of feigned symptoms for secondary gain, and the implication of stigma in an African American young male arrested for a drug-related crime.

## 1. Introduction

Catatonia is a syndrome of neuropsychiatric symptoms with observable motor and behavioral disturbances. Features may include mutism, staring, immobility, posturing, grimacing, rigidity, negativism, and withdrawal, among others [1]. While catatonia most often falls under the umbrella of psychiatry, other specialties may have different terminology to describe the same phenomenon, such as “acute hypothalamic instability,” “minimally conscious state,” or “paroxysmal sympathetic hyperactivity.” It may exist on a continuum or with discrete subtypes of hypo-, hyper-, or parakinetic (odd or bizarre) movements or activity [2]. In addition to a diverse array of clinical manifestations, a universally accepted set of diagnostic criteria is absent, which may make it hard to recognize. Furthermore, though the signs of catatonia have been well defined, the definitions of what constitutes the full catatonia syndrome vary between authors. The DSM-V specifies that only three of the following criteria must be met to reach a diagnosis of catatonia–catalepsy, waxy flexibility, stupor, agitation, mutism, negativism, posturing, mannerisms, stereotypies, grimacing, echolalia, and echopraxia. By contrast, the Bush–Francis Catatonia Rating Scale is a standardized instrument used to detect and grade the severity of catatonia that includes a broader range of 23 features such as autonomic instability, gegenhalten, ambitendency, and automatic obedience, among others.

Historically, catatonia was considered a form of schizophrenia, but DSM-IV recognized it as a separate entity that is characterized by its psychomotor symptoms and can accompany a wide array of medical and psychiatric conditions. Potential etiologies of the catatonic syndrome are vast and include primary psychiatric illness (major depression, bipolar disorder, schizophrenia) as well as other medical conditions that often present with neuropsychiatric symptoms such as autoimmune or paraneoplastic encephalitis, systemic lupus erythematosus, epilepsy, brain infection, intracranial lesions, and drug intoxication or withdrawal [3]. Neuroimaging and blood flow studies have implicated the motor system within the supplemental and premotor cortex in catatonia—neurocircuitry structures that are involved in the preparation and execution of movement [4]. Of note, there are also genetic implications to the development of catatonia, with first-degree relatives demonstrating increased rates of affective disorders as well as catatonic symptoms and increased syndrome severity [4].

The clinical recommendations for the management of catatonia are to first have a high index of suspicion for its presence and utilize rating scales to confirm the diagnosis. Early recognition and treatment are important, as is providing prophylaxis and medical support to prevent potentially life-threatening complications of immobility. Benzodiazepines, particularly lorazepam, have long been recognized as the gold standard treatment in acute catatonia (sublingually, intravenously, or intramuscularly), and if necessary, electroconvulsive therapy can be used [4]. Investigation continues into alternative treatment approaches such as glutamate agonists (amantadine or memantine) or rTMS [5].

The body of literature reporting catatonia secondary to traumatic brain injury is limited. Many cases were marked by a delayed diagnosis, and some presented with catatonia months to years after the initial injury [6–8]. Here, we present a case of catatonia secondary to traumatic brain injury with delayed recognition likely due to biases related to the social context of his presentation.

## 2. Case Presentation

A black male in his early 20s with a remote history of depression as an adolescent and current cannabis use presented following an assault while incarcerated. He was found unconscious with mental status changes and brought to the hospital. Initial examination and work-up revealed right-sided periorbital edema and ecchymosis. The patient was arousable to noxious stimuli but would quickly return to sleep and was not interactive during multiple examination attempts. The emergency department staff believed there was a volitional component to his lack of participation in the exam, as he would push away from providers as they examined him or lie unresponsive. At one point, the patient was able to ambulate briefly while in the emergency department (although he ran into several objects) and tolerated an oral intake challenge with juice. Over the course of 5 days, the patient was returned to the emergency department by the facility four more times due to concerns that his mental status was not improving. The facility reported the patient was unable to attend to his ADLs. He was seen to be withdrawn, not eating or drinking, and toileting on himself or the floor. Additionally, guards (as he was incarcerated for part of his hospital admission) reported that he would occasionally speak to family members and staff. However, when he was offered paperwork to sign himself out of jail with an ankle monitor, he only gazed at the officer without speaking.

Urine toxicology was positive for THC and a negative blood alcohol level. He was found to have ketones consistent with starvation ketosis. Lumbar puncture revealed CSF cell count with six total nucleated cells present, <2,000 RBC, 54 mg/dL glucose, and 39 mg/dL protein. Meningitis/encephalitis panel for 14 common viral and bacterial pathogens and antibody testing for West Nile Virus was negative. CT of the head showed no fractures of the face/vertebra and was negative for other acute findings. EEG was read as consistent with a normal drowsy state. MRI of the brain, both with and without contrast, showed a cerebellar developmental venous anomaly but no acute findings. A neurology consult was obtained. His neurologic exam was noted to be variable, with poor participation despite repeated prompting, making full examination difficult. He responded to pain equally in all extremities, demonstrated no abnormal movements, had 2+ reflexes symmetrically, and normal pupillary response. Neurology ultimately felt that his LP, MRI, and EEG were unremarkable, and his symptomatology could be explained by postconcussive symptoms.

Once the patient was admitted, he was initially treated with a trial of Modafinil for suspected postconcussive syndrome, to which there was no response. On Day 6 of his hospitalization, when the psychiatric consultation service reconsidered his disordered behavior, psychomotor retardation, and speech with consideration for the presence of catatonia, the question of “volitional” inactivity took on a new meaning. There were numerous catatonia signs present to variable degrees: mutism, decreased oral intake, negativism, withdrawal, and decreased spontaneous movement. No automatic obedience, ambitendency, or rigidity was appreciated. A presumptive diagnosis of catatonia was made, and a trial of lorazepam 2 mg IV was initiated. Fifteen minutes following administration, the patient was talking fluently, requested food, and leapt out of bed. He exhibited purposeful movement and was more interactive, particularly with family at the bedside, to provide encouragement. He was maintained on a dose of lorazepam 1 mg three times daily, which was later titrated down to 0.5 mg nightly by the day of discharge due to concerns that this was contributing to sedation. Oral intake was recorded by nursing staff as improved, with the patient eating 0% of meals upon arrival and 100% by the time of discharge. Daily weights were not recorded. Physical therapy recommended placement for rehabilitation due to poor safety awareness, delayed processing/sequencing, and impaired coordination. The patient declined this option in favor of discharge home with home health and outpatient therapy services. He was seen in the outpatient setting 2 weeks after discharge with worsened symptoms of catatonia, which responded to increased lorazepam dosing of 2 mg 3 times daily. His symptoms improved, and his benzodiazepines were weaned over the next 2 months. Ultimately, he was lost to follow-up as he missed appointments and could not be reached via telephone or the electronic medical record patient portal to reschedule.

## 3. Discussion

Catatonia may be underrecognized in the general medical population due to a lack of training in its etiology, presentation, and treatment. Workup has been demonstrated to be more limited when a psychiatric illness is suspected as opposed to a differential diagnosis that includes delirium and other neurologic and systemic causes [9]. Because the etiology of conditions that can cause catatonia is so vast, differential diagnoses should remain broad while investigative laboratory analysis is undertaken to rule out reversible causes. Immune and antibody-mediated conditions have often been implicated, such as autoimmune or paraneoplastic encephalitis, systemic lupus erythematosus, multiple sclerosis, and autoimmune thyroiditis, though no single unifying immunologic mechanism has been elucidated [10]. Other brain conditions, such as seizures, stroke, dementia, and CNS infections, have also presented with catatonic symptoms, positing a theory that disruption in certain neural circuitry may be responsible for the manifestation of catatonia. Both GABA agonist withdrawal and intoxication have been implicated in the development of catatonia, suggesting dysfunction in the GABAergic neurons of cortico-striatal-thalamocortical motor loops [3, 11]. Walther et al. [4] have further explored the structural and neural mechanisms of catatonia with additional recent evidence implicating dysfunctional motor networks.

There was unfortunately a delay in diagnosis and treatment of catatonia in this case, likely due in part to stigma from incarceration and drug use. His mental status changes were initially attributed to postconcussive syndrome and potential intoxication from synthetic cannabinoids. Additionally, the patient's negativism was interpreted as a voluntary resistance to examination and poor effort rather than a feature of illness. As such, it was felt that he presented with the intention of secondary gain of release from his situation. Only after several days of hospitalization did this suspicion fall away, as he was offered paperwork for release that he did not sign or even meaningfully acknowledge. Ultimately, it was a shift in team coverage on the psychiatric consultant service that led to the recognition and treatment of catatonia.

Cognitive and affective biases can interfere with medical decision-making, and in the case of a young black male who was incarcerated due to drug-related crimes, stigma and racial biases may have played a role in the initial impression of malingering. This has previously been reported by Franklin et al. [12]. The limited cases of reported TBI-related catatonia have all benefited from intervention with benzodiazepines or a related medication, such as zolpidem, in addition to medically supportive interventions.

## 4. Conclusion

Catatonia is an urgent, life-threatening condition that is treatable when recognized and treated. Personal biases can result in misinterpretation of physical findings, leading to a delay in diagnosis. We recommend vigilance to consider the diagnosis of catatonia, particularly with atypical or nonspecific features such as autonomic instability and withdrawal or stupor.

## Data Availability

There are no identifiable data associated with this report.
